# Jingtong Granule: A Chinese Patent Medicine for Cervical Radiculopathy

**DOI:** 10.1155/2015/158453

**Published:** 2015-05-12

**Authors:** Liguo Zhu, Jinghua Gao, Jie Yu, Minshan Feng, Jinyu Li, Shangquan Wang, Xu Wei

**Affiliations:** ^1^Department of Spine, Wangjing Hospital, China Academy of Chinese Medical Sciences, Huajiadi Street, Chaoyang District, Beijing 100102, China; ^2^Department of Orthopaedics, Dongzhimen Hospital, Beijing University of Chinese Medicine, No. 5 Haiyuncang Street, Dongcheng District, Beijing 100700, China; ^3^Department of General Orthopedics, Wangjing Hospital, China Academy of Chinese Medical Sciences, Huajiadi Street, Chaoyang District, Beijing 100102, China; ^4^Department of Scientific Research, Wangjing Hospital, China Academy of Chinese Medical Sciences, Huajiadi Street, Chaoyang District, Beijing 100102, China

## Abstract

*Objective*. This paper systematically assessed the efficacy and safety of Jingtong granule (JG) for cervical radiculopathy (CR).* Methods*. Randomized controlled trials comparing JG with no intervention, placebo, or conventional therapies were retrieved. The trials testing JG combined with conventional therapies versus conventional therapies were also enrolled. Study selection, methodological assessment, data extraction, and analysis were conducted in accordance with the Cochrane standards. The strength of evidence was evaluated according to GRADE approach.* Results*. Three trials with 400 participants were included. Methodological quality was evaluated as generally low. One study found that JG showed significant difference on decreasing pain scores compared with placebo. Meta-analysis indicated that JG plus conventional analgesic exhibited a significant immediate effect on the pain scores (WMD = 1.63; 95% CI: 1.29 to 1.98; *P* < 0.00001). Additionally, JG combined with analgesic presented beneficial immediate effect on neck disability index. However, the treatment effects of JG demonstrated in the trials were not large, and the safety of JG was unproven. Finally the evidence level was evaluated to be low.* Conclusions*. Our results indicated that JG showed some potential benefits for CR. Nevertheless, treatment effects are uncertain due to both the methodological concerns and the very modest reported improvements.

## 1. Introduction

Cervical radiculopathy (CR) is a common condition typically characterized by neck pain and radiating arm pain or hypoesthesia or motor dysfunction in the neck and upper limbs [1–3]. Neck pain with radicular symptoms is most frequently reported to occur in middle-aged and elderly people. The annual incidence is 2.1 cases per 1000 in the age group of 50–54 years [4]. Most of patients suffering from CR present low performance in neck active range of motion, neck muscle endurance, or hand grip strength [5]. Accordingly, the impingement is also a significant source of disability and associated with large healthcare costs especially for those who will undergo surgery [6, 7].

Pain management is a fundamental and effective intervention for relieving the radicular symptoms and improving quality of life. Nonoperative treatments have been widely accepted and often used to help control nerve root pain [3, 8, 9]. Among them, oral anti-inflammatory drugs or epidural steroid injections as an analgesic are regularly used to treat pain [10–12]. However, the latest evidence-based guideline released by the North American Spine Society (NASS) in 2011 is unable to make recommendations regarding the effectiveness of pharmaceutical treatments for CR due to lack of well-designed trials [13]. Another major problem for medication as a therapeutic strategy is the many important side effects of conventional drugs [14]. Simultaneously, all of the adverse events are commonly seen in the fourth or fifth decade of life in which there is a peak of CR [15]. Traditional Chinese medicine (TCM), as one of the complementary and alternative therapies, has been well recognized in relieving symptoms of neck pain [16–18]. It is also found out that an increasing number of randomized trials and systematic reviews are used to assess the effectiveness and safety of various TCM modalities in improving the neck pain associated with CR [19, 20]. In addition, more and more physicians or patients firstly select the TCM therapy for better safety in China. Among the TCM modalities, Chinese herbal medicine is becoming increasingly popular and frequently used for the treatment of CR.

According to the theory of traditional Chinese medicine, the main cause of CR has been attributed to qi stagnation and blood stasis (a presentation of TCM syndrome) [21]. Jingtong granule (JG), a Chinese oral patent medicine, has been approved and recommended by China Food and Drug Administration (CFDA) for treating CR [22]. JG is comprised of seven commonly used herbs, including* Radix notoginseng* (Sanqi),* Rhizoma Ligustici Chuanxiong* (Chuanxiong),* Rhizoma Corydalis* (Yanhusuo),* Radix Paeoniae Alba* (Bai Shao),* Radix Clematidis* (Wei Ling Xian),* Radix Puerariae* (Gegen), and* Rhizoma et Radix Notopterygii* (Qiang Huo). The experimental research has shown that JG is an effective drug in alleviating congestion, edema, and lymphocyte infiltration of inflammatory reaction, lowering the amount of substance P, reducing the proliferation of fibroblasts and collagen fibers and cicatrization of the nerve roots, and eventually promoting the recovery of nerve function [23–25].

In recent years, clinical trials have been widely reported for the application of JG in patients with CR and published in the Chinese medical literatures [26–28]. Nevertheless, there is no critically appraised evidence such as a systematic review to evaluate clinical efficacy and safety of JG for CR. This review aims to assess current evidence of JG for CR from randomized controlled trials (RCTs).

## 2. Methods

### 2.1. Inclusion Criteria and Exclusion Criteria

All the parallel RCTs testing the efficacy of JG for the treatment of CR were enrolled. RCTs based on JG compared with no treatment, placebo, or conventional therapies were considered. Combined therapy of JG and conventional analgesic compared with conventional analgesic was also retrieved. The primary outcome measures included pain score changes in internationally recognised pain-related assessment tool. The secondary outcomes analysed in this review were neck disability index (NDI) or quality of life. The timing of outcome assessment was divided into four time periods: immediately after treatment (up to one day), short-term follow-up (between one day and three months), intermediate-term follow-up (between three months and one year), and long-term follow-up (one year and beyond) [29–31]. Quasi-randomized controlled trials were not included. Multiple publications reporting the same groups of participants were excluded.

### 2.2. Search Strategies and Study Selection

The following seven electronic databases were searched from their respective inception through 14 February 2015: EMBASE, PubMed, Cochrane Library, Chinese Biomedical and Medical Database (CBM), Chinese National Knowledge Infrastructure (CNKI), Chinese Scientific Journal Database (VIP), and Wanfang Database. The search terms included “cervical radiculopathy,” “cervical spondylotic radiculopathy,” and “cervicobrachial pain” combined with “Jingtong granule.” No restriction on publication status or language was imposed. Two authors (X. Wei and J. Y. Li) independently searched and selected the RCTs according to the inclusion criteria. Disagreements were resolved by discussion between the authors. The bibliographies of the included studies were also searched for additional references.

### 2.3. Data Extraction and Methodological Quality Assessment

Two reviewers conducted data extraction (X. Wei and J. Yu) separately according to the preset contents. The extracted data included first author names, year of publication, sample size, population characteristics (age of patients and duration of symptom), intervention characteristics, duration of treatment, outcome assessment, and overall conclusions about the effectiveness of JG. Intervention and control group details included drug, medication doses, therapeutic regimen, and treatment duration. The data was entered into an electronic database by the two reviewers independently. Differences were resolved by discussion and reached consensus through a third reviewer (J. H. Gao).

The methodological quality of included studies was assessed independently in accordance with the criteria from the Cochrane Handbook for Systematic Review of Interventions (X. Wei and J. Yu) [32]. The domains included random sequence generation, allocation concealment, blinding of participants and personnel, blinding of outcome assessment, incomplete outcome data, selective reporting, and other bias. All the trials were categorized into three levels: low risk of bias, high risk of bias, and unclear risk of bias.

### 2.4. Data Synthesis

We used Review Manager 5.2.0 software (Cochrane Collaboration) to conduct data analysis. Since all outcomes were continuous data, weighted mean difference (WMD) was used to assess the difference between experimental group and comparison group. And the 95% confidence intervals (CI) were calculated in the meta-analysis. Subgroups analysis was performed among different types of comparisons (including JG versus inactive therapy and JG plus active drugs versus active drugs). Heterogeneity of effect sizes was assessed with the *I* ^2^ statistic. Pooled-effect estimate was calculated using random-effect model if substantial heterogeneity existed (*I* ^2^ > 50%), whereas the fixed-effect model was used to analyse data that were not significantly heterogeneous. Funnel plot analysis would be performed to assess publication bias if there were sufficient clinical trials.

### 2.5. Qualitative Analysis of Evidence Level

The grading of recommendations assessment, development, and evaluation (GRADE) approach was used to evaluate the evidence levels for the outcomes in the meta-analysis. It specified four levels: high, moderate, low, and very low quality evidence [33–35]. Two investigators (X. Wei and J. Yu) extracted data from included trials. Differences were resolved by a third reviewer (L. G. Zhu).

## 3. Results

### 3.1. Description of Included Trials

**Figure 1:**
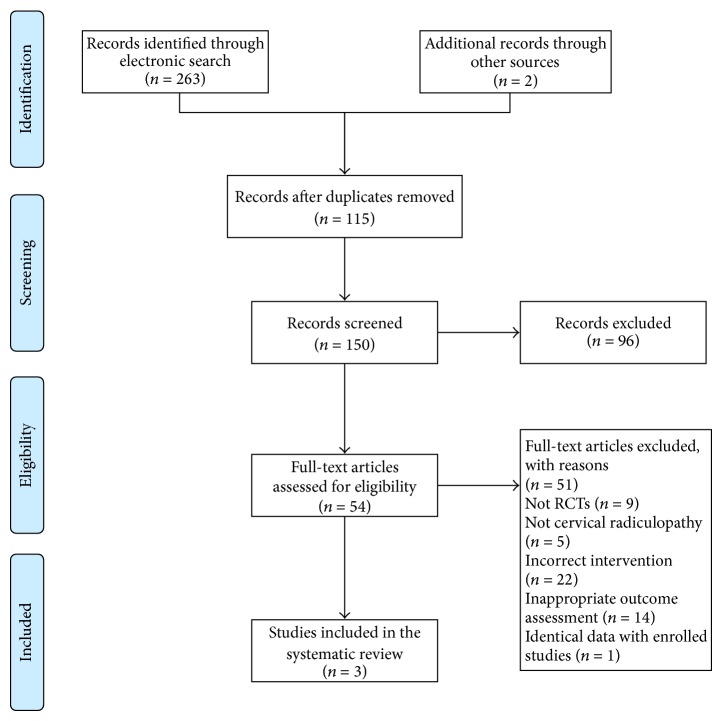
PRISMA 2009 flow diagram.

The search in electronic databases yielded 263 articles, and two studies were identified in retrieving other relevant sources. First of all, 115 duplicated articles were excluded. Secondly, 54 articles were identified during title and abstract screening. Then, the full texts of 54 trials were retrieved for further eligibility evaluation and a total of four studies met the inclusion criteria. The reasons for exclusion were as follows: not RCTs (*n* = 9), not cervical radiculopathy (*n* = 5), incorrect intervention (*n* = 22), and inappropriate outcome assessment (*n* = 14). One article published in 2013 [36], however, was later ruled out because the extracted data including mean difference and standard deviations was much the same as the enrolled article by Liu and Zhang in 2008 [37]. Therefore, three studies were included in our review.  Figure 1 depicted the literature screening process. All RCTs were conducted in China and published in Chinese [37–39]. They were published between 2008 and 2013.

### 3.2. Essential Characteristics of Included Trials

Characteristics of the enrolled RCTs in the interview were summarized in Table 1. Only one study compared JG with placebo [37], and the other two studies compared JG plus conventional analgesic (mannitol, dexamethasone, and ibuprofen codeine sustained tablets) with conventional analgesic alone [38, 39]. The sample size ranged from 120 to 160 with a total size of 400. All patients were adults (≥18 years), and the duration of disease varied from 3 hours to 5 years. The oral dose of JG was 4 g every time in all trials, but patients in two trials were administrated three times a day [37, 38] and the rest was administrated once a day [39]. The duration of treatment in the included studies was within 1 month. Visual analogue scale (VAS) or NDI scores were used as outcome assessment indexes. To our regret, adverse events were not reported in all identified studies.

### 3.3. Methodological Quality of Included Trials

**Figure 2:**
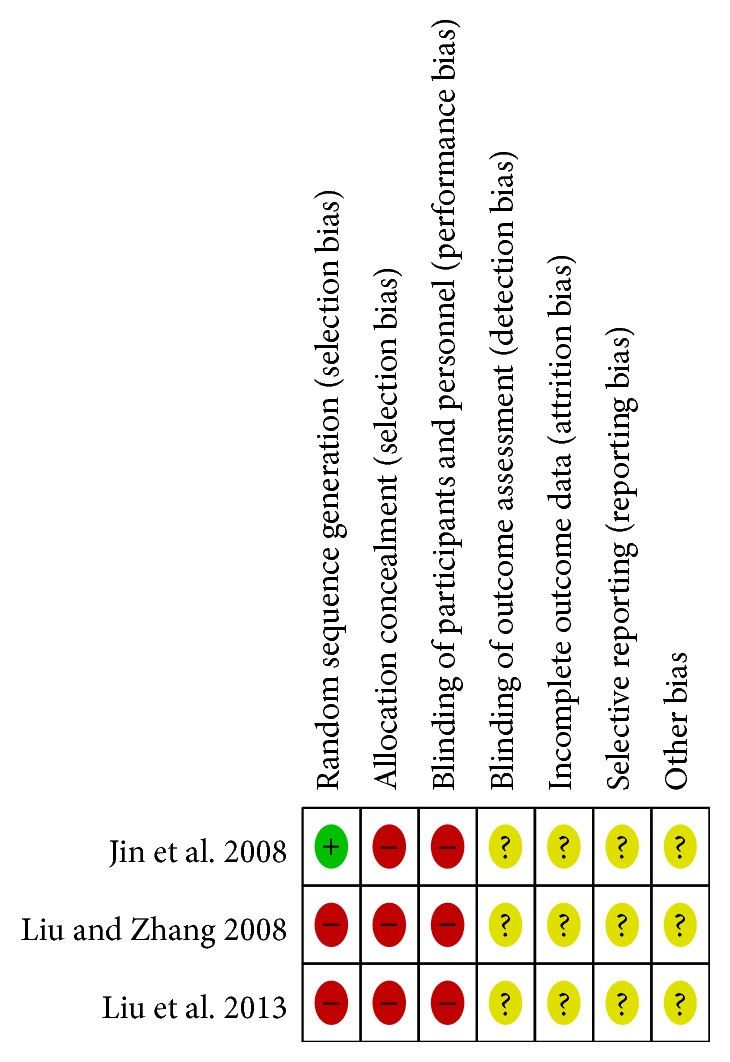
Risk of bias summary: review authors' judgements about each risk of bias item for each included study. Yellow (?): unclear risk of bias; green (+): low risk of bias; red (−): low risk of bias.

**Figure 3:**
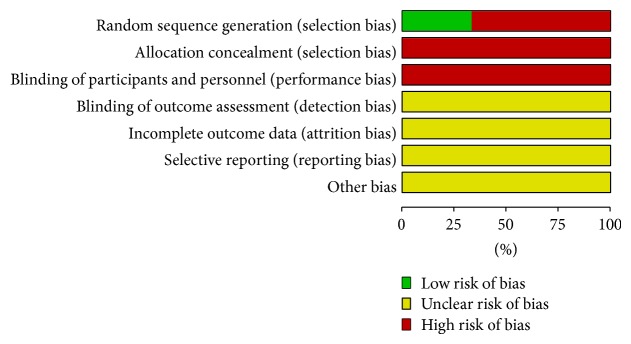
Risk of bias graph: review authors' judgements about each risk of bias item presented as percentages across all included studies.

The assessment of methodological quality in included studies was represented in Figures 2 and 3. The quality of reporting was generally poor, and all the trials were high risk of bias. Of the three studies, only one [38] reported the random sequence generated from a random number table, and the others mentioned “patients were randomly allocated” by registration order. Obviously, the randomization method of registration order failed to reach the requirements of random assigning. None of the studies used allocation concealment or blinding of participants and personnel although the placebo was performed [37]. No trial explicitly described blinding of outcome assessment. Additionally, incomplete outcome data could not be evaluated due to insufficient information. Dropout and withdrawal data were not provided for the trials. None of the trials had a pretrial estimation of sample size. We could not decide whether selective reporting or other important risks of bias existed as no preregistered protocols could be obtained.

### 3.4. Effect of the Interventions

Table 1 provided detailed information for the intervention and control group. All the trials focused on the effects of JG on CR. On the other hand, minimum clinically important difference (MCID) was used to assess the treatment effect and generally considered to be 10 on a 100-point pain intensity scale [40–43]. For the pain measures, the effect was assumed to be small when it was less than 10% of the VAS scores, medium when it was between 10% and 20% of the VAS scores, and large when it was from 20% to 30% of the VAS scores [31]. For the NDI scores, we used a minimum clinically important difference of up to 10/50 for CR [44].

#### 3.4.1. JG versus Placebo

Only one trial compared the clinical efficacy of JG as monotherapy with placebo for CR [37]. The primary outcome measure was VAS scores ranging from 0 to 100. Meta-analysis suggested that JG showed significant difference on decreasing VAS scores (WMD = 18.55; 95% CI: 11.82 to 25.24; *P* < 0.00001). The treatment effect of JG in the trial was considered to be medium.

#### 3.4.2. JG Plus Conventional Analgesic versus Conventional Analgesic

**Figure 4:**
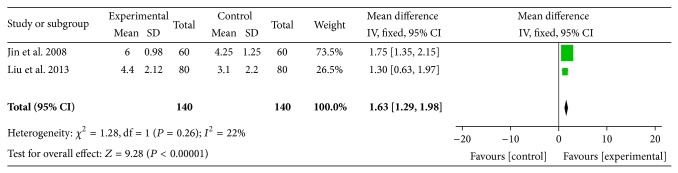
Forest plot of comparison: JG plus conventional analgesic versus conventional analgesic and outcome: VAS scores.

The effectiveness of JG plus conventional analgesic versus conventional analgesic alone was evaluated in two trials [38, 39]. The primary outcome measure was VAS scores ranging from 0 to 10. Meta-analysis (Figure 4) of these two trials indicated that JG plus conventional analgesic exhibited a significant pain-relieving immediate effect on the VAS scores (WMD = 1.63; 95% CI: 1.29 to 1.98; *P* < 0.00001) and without significant heterogeneity (*χ* ^2^ = 1.28, *P* = 0.26; *I* ^2^ = 22%). Fixed-effect model was used to calculate pooled-effect estimate. The treatment effects of JG in two trials were considered to be medium.

One RCT performed by Liu et al. [39] reported the immediate effect of JG combined with conventional analgesic (ibuprofen codeine sustained tablets) on NDI scores compared with conventional analgesic alone. The meta-analysis showed there was significant beneficial effect on the combination group (WMD = 8.40; 95% CI: 7.47 to 9.33; *P* < 0.00001). The treatment effect of JG in the trial was considered to be small.

### 3.5. Funnel Plot Analysis

Funnel plot analysis could not be conducted due to the small number of included studies (less than 10) in the meta-analysis.

### 3.6. Grading of Evidence Level

According to the GRADE method, two basic factors including limitations in study design and highly suspected publication bias for the outcome decreased the quality of evidence in the meta-analysis. However, none of the items such as large magnitude of effect, all plausible confounding, and high dose-response gradient upgraded evidence level of the result. Therefore, the evidence level was evaluated to be low.

## 4. Discussion

### 4.1. Summary of Evidence

CR is a modern epidemic which has the highest incidences of neck pain, upper back pain, and wrist and hand weakness, affecting most middle-aged and elderly people at some time during their lives [45]. Although western medicine offers many options for the management of neck pain such as CR, limited evidence and undesirable side effects have given rise to a dramatic increase in the use of complementary and alternative medicine and an increase in interest in Chinese herbs [46, 47]. Chinese herbs, including Chinese oral patent medicine, have been used in China for many years to treat diseases [48]. The clinical practice of JG for CR is a case in point. The biological effects of JG have been well described by Zhang et al. [23–25]. While several clinical trials reported JG for CR, there was no systematic review specially dealing with its effectiveness and safety in the treatment of CR. So this is the first review and meta-analysis to provide an evidence-based evaluation of JG for the management of CR from 3 RCTs with a total of 400 participants.

JG or combined therapy of JG and conventional analgesic presented statistically significant benefit in VAS and NDI scores as compared with placebo (*n* = 1) or conventional analgesic alone (*n* = 2). When used alone, JG was found to be beneficial for the reduction of VAS scores (WMD = 18.55; 95% CI: 11.82 to 25.24; *P* < 0.00001) in immediate effect compared with placebo. When used in combination with analgesic, the pooled effect suggested that JG plus conventional analgesic had significantly higher VAS scores changes (WMD = 1.63; 95% CI: 1.29 to 1.98; *P* < 0.00001; *I* ^2^ = 22%) in immediate treatment than those treated with conventional analgesic alone. Moreover, JG plus ibuprofen codeine sustained tablets was found to be effective in terms of improving NDI scores (WMD = 8.40; 95% CI: 7.47 to 9.33; *P* < 0.00001) when compared with ibuprofen codeine sustained tablets. However, the treatment effects of JG demonstrated in the trials were not large according to the MCID. In addition, no extra information about adverse events was available to assess the adverse events of JG. Therefore, it is difficult to draw a definite conclusion regarding the safety of JG.

The quality of evidence level according to the GRADE approach was assessed to be low in the meta-analysis. For the VAS scores, the main reasons for downgrading evidence level were poor study design/execution and likely potential publication bias. As the total sample size of the two studies included in the meta-analysis was small, we were unable to acquire large magnitude of effect in the treatment of CR.

### 4.2. Limitations

The following limitations should be noted before accepting the findings of this review. All included trials were prone to some methodological issues and potential risk of bias, which could directly weaken the strength of recommendation.

Firstly, only one trial claimed random sequence allocated by random number table, whereas the other two trials used patients registration order as random method. Obviously, inappropriate random method such as registration order was not recommended. None of the studies reported allocation concealment. So selection bias might have occurred.

Secondly, none of the enrolled studies described double blind method as well as the blinding of outcome assessment. In this review, although a randomized placebo-controlled clinical trial was conducted by Liu et al., the medication dosage of JG (4 g/time) appeared somewhat different from placebo (300 mg/time). In addition, we were unable to acquire preparation method of placebo by contacting the original authors. As for outcome assessment, patient-reported outcome such as VAS scores might have certain subjective [49, 50]. Therefore, performance bias and detection bias might be generated in the conclusion.

Thirdly, neither withdrawals nor drop-outs were reported in each study. We did not identify whether incomplete outcome data existed. Thus, attrition bias in this study was still unclear. As the study protocols were not published publicly or registrated through the website of Chinese clinical trial registry (http://www.chictr.org/en/) or international clinical trial registry by U.S. national institutes of health (http://clinicaltrials.gov/) and so forth, this could lead to an unclear risk of reporting bias. Thus, we suggest that researchers of RCTs publish complete and clear protocol in the future. Furthermore, a funnel plot was not available to check for possible publication bias for those outcomes due to the limited number of included studies. Publication bias might exist in the results. Some other bias, for instance, whether implementing intention-to-treat analysis or not, was also nondeterministic condition.

Last but not least, multicenter and large scale clinical study design was not applied in all of the included trials. The outcomes from the enrolled RCTs are mainly VAS scores and only one study mentioned NDI scores. However, quality of life was also recommended as an important outcome for evaluating the treatment effect of CR in the guideline issued by the North American Spine Society [13]. What is more, only immediate effect was observed in the review but short-term and long-term follow-up effect remained unknown due to lack of follow-up. Therefore, it needs to be designed and reported appropriately in the future clinical trials. In addition, we should ensure rational application of drug based on medicine specification in clinical practice.

### 4.3. Conclusion

Although this systematic review suggested some benefits of JG for CR patients, the recommendation of findings was limited due to the poor quality of previous studies. Additionally, because of the limited number of included RCTs in this subgroup, further clinical evidence is needed to confirm these conclusions.

## Figures and Tables

**Table 1:** Summary of the included studies in the review.

First author (year)	Sample size (T/C)	Population characteristics	Intervention	Comparison	Duration of treatment	Outcome assessment	Conclusion
Liu, 2008 [37]	120 (84/36)	Age: 21 to 60 (T/C: NA) Course: NA	JG (4 g, Tid, PO)	Placebo (300 mg, Tid, PO)	T: 28 days C: 28 days	VAS (0 to 100) Diff: 18.53	The results suggested JG was better than placebo for reductions of pain in the treatment of CR.

Jin, 2008 [38]	120 (60/60)	Age: 27 to 59 (T/C: NA) Course: 3 hours to 5 days (T/C: NA)	JG (4 g, Tid, PO) + C	First three days: 20% mannitol (250 mL, Ivgtt, Qd) + dexamethasone (10 mg, Ivgtt, Qd) After three days: 20% mannitol (250 mL, Ivgtt, Qd) + dexamethasone (5 mg, Ivgtt, Qd)	T: 6 days C: 6 days	VAS (0 to 10) Diff: 1.75	The result of randomized controlled clinical trial showed that both JG and western medicine therapies were effective and superior to single western medicine in relieving pain for CR.

Liu, 2013 [39]	160 (80/80)	Age: 26 to 66 (T: 26 to 66; C: 30 to 65) Course: 3 months to 5 years (T/C: 3 months to 5 years)	JG (4 g, Qd, PO) + C	ICST (2 pills/time, Bid)	T: 14 days (JC) C: 5 days	VAS (0 to 10) Diff: 1.30 NDI Diff: 8.40	The improvements in VAS and NDI scores difference of intervention group were better than those of the control group.

T: treatment group; C: control group; Diff: difference between before and after treatment in both groups; NA: not reported.

JG: Jingtong granule; ICST: ibuprofen codeine sustained tablets.

Tid: three times a day; Bid: twice a day; Qd: once a day; PO: oral administration; Ivgtt: intravenous guttae.

VAS: visual analogue scale; NDI: neck disability index; CR: cervical radiculopathy.
